# Protective role of p53 in skin cancer: Carcinogenesis studies in mice lacking epidermal p53

**DOI:** 10.18632/oncotarget.7897

**Published:** 2016-03-03

**Authors:** Angustias Page, Manuel Navarro, Cristian Suarez-Cabrera, Josefa P. Alameda, M. Llanos Casanova, Jesús M. Paramio, Ana Bravo, Angel Ramirez

**Affiliations:** ^1^ Molecular Oncology Unit, Centro de Investigaciones Energéticas, Medioambientales y Tecnológicas (CIEMAT), Madrid, Spain; ^2^ Biomedical Research Institute I+12, University Hospital “12 de Octubre”, Madrid, Spain; ^3^ Department of Veterinary Clinical Sciences, Faculty of Veterinary Medicine, University of Santiago de Compostela, Lugo, Spain

**Keywords:** p53, transgenic mice, papilloma, skin SCC, DMBA/TPA

## Abstract

p53 is a protein that causes cell cycle arrest, apoptosis or senescence, being crucial in the process of tumor suppression in several cell types. Different *in vitro* and animal models have been designed for the study of p53 role in skin cancer. These models have revealed opposing results, as in some experimental settings it appears that p53 protects against skin cancer, but in others, the opposite conclusion emerges. We have generated cohorts of mice with efficient p53 deletion restricted to stratified epithelia and control littermates expressing wild type p53 and studied their sensitivity to both chemically-induced and spontaneous tumoral transformation, as well as the tumor types originated in each experimental group. Our results indicate that the absence of p53 in stratified epithelia leads to the appearance, in two-stage skin carcinogenesis experiments, of a higher number of tumors that grow faster and become malignant more frequently than tumors arisen in mice with wild type p53 genotype. In addition, the histological diversity of the tumor type is greater in mice with epidermal p53 loss, indicating the tumor suppressive role of p53 in different epidermal cell types. Aging mice with p53 inactivation in stratified epithelia developed spontaneous carcinomas in skin and other epithelia. Overall, these results highlight the truly protective nature of p53 functions in the development of cancer in skin and in other stratified epithelia.

## INTRODUCTION

The gene coding for cellular tumor protein p53, *TP53*, is the most frequently mutated gene in human cancers, being altered in approximately 50% of malignancies (for a review, see [1]). p53 is a transcriptional factor able to interact both with DNA, regulating the expression of a myriad of target genes, and with numerous proteins, mutually modifying their activity. p53 is usually found at very low levels in the cells, but it accumulates in case of genetic damage. p53 promotes multiple cell functions associated to tumor suppression, as cell cycle arrest, apoptosis and cellular senescence, being fundamental in the prevention of the division of cells that have suffered DNA damage; for all these functions, p53 has been nicknamed as “guardian of the genome” [2]. p53 also precludes tumor formation by regulating additional mechanisms, as genetically modified mice expressing a mutant form of p53 unable to direct cell cycle arrest, apoptosis and senescence do not suffer from early onset tumor formation [3]. Among these additional mechanisms of cancer prevention mediated by p53 are regulation of DNA stability, cellular metabolism, autophagy, stem cell maintenance and metastasis (reviewed in [4]). The cancer preventive activity of p53 could also be mediated by other pathways; for example, it has been shown that rapamycin, a known inhibitor of mTOR pathway, decreases the number of tumors originated in p53^−/−^ or p53^+/−^ mice [5, 6], suggesting a causative role of mTOR inhibition in tumor prevention. As p53 induces the expression of several negative regulators of mTOR pathway [7], part of the antitumoral effect of p53 could be mediated by mTOR downregulation.

Despite the intensive studies performed in the last 35 years, we are far from having a precise knowledge of p53 functions. The complete comprehension of p53 roles is hindered by the multiplicity and complexity of the biological processes it regulates. So, besides its role in cancer, p53 is implicated in clearance of apoptotic cells and prevention of autoimmune diseases [8], as well as in embryonic development, resulting both its inactivation and its hyperactivation in developmental defects [9, 10]. Another source of complexity comes from the fact that some functions of wild type (wt) or mutant p53 are not equally regulated in different tissues or cell types [11]. In addition, there are more than ten isoforms transcribed from the *TP53* gene, with important differences in subcellular location and biological activity among them, and scarcely known cross-regulation relationships. Illustrative of this complexity is the recent discovery of a p53 isoform with a surprising role in metastasis promotion [12]. So new studies and experimental models are required for deepening in the understanding of p53 functions in cell physiology and tumor transformation.

Mutation of p53 can result in cancer by the effects associated to loss of its normal functions or by the acquisition of new transforming capabilities, acting as a dominant-negative mutant of the wild type p53 or as a *bona fide* oncogene with transformation potential [13]. The presence and the type of p53 mutations in tumors are clinically important, as mutated versions of p53 can greatly modify the response to therapies [14].

Many animal models have been developed for the study of p53. Knockout mice lacking p53 die at early age and develop an array of spontaneous tumors, mainly lymphomas and sarcomas, with different frequency depending on the genetic background [15, 16]. More recently, after the generation of p53 floxed (fl) mice [17], a plethora of studies have been performed by using animal models with p53 inactivation restricted to specific cell types. Transgenic (Tg) mouse models bearing different mutant forms of p53, alone or in association with mutations in other genes, have also rendered relevant information about the role of p53 in tumor transformation in different types of cancer [18].

Skin cancer is the most abundant human oncogenic lesion, and *TP53* is often mutated in patients with skin cancer [19]. Different spectra of *TP53* mutations have been found in different skin tumor types (i.e., basal cell carcinoma (BCC), squamous cell carcinoma (SCC), and melanoma) [20].

Data in the literature indicate that p53 cooperates with a plethora of oncogenic insults, such as Ras activation [21], Rb inactivation [22], loss of αv integrin [23] or Snail overexpression [24], in the development of skin carcinomas. Indeed, a number of experiments performed in different experimental models indicate a tumor suppressor role of p53 in skin cancer. So p53 null mice are prone to the development of skin SCCs after UV light radiation [25], and a requirement for p53 loss for malignant conversion of skin tumors has been described, even in the context of various other genetic oncogenic insults [26]. In addition, mice lacking p53 in epidermis develop spontaneous skin tumors [22, 23, 27]; interestingly, skin and tumors lacking p53 showed higher chromosomal instability than those with p53^wt^ background [28, 29]. In a different cancer model (i.e., mouse keratinocytes grafted onto nude mice), aimed to study the cooperation of p53 loss and v-ras^Ha^-mediated initiation, the tumor originated showed increased growth and malignancy in the absence of one or two p53 alleles [30]. Altogether, these results seem to demonstrate a protective role of p53 in skin carcinogenesis and malignization.

But there are also reports indicating an apparently discrepant protumoral role of p53 in skin carcinogenesis. So two-stage skin carcinogenesis experiments show that p53 null mice develop fewer and smaller skin tumors than p53 wild type mice [31, 32], indicating that the presence of p53 in skin cells provokes the emergence of more tumors, at difference with the results found in epithelial cells of the intestine [33] and other cell types. Other experimental settings have also led to the notion of an oncogenic role of p53 in skin cancer; so p53 loss precludes tumor formation in transgenic mice overexpressing activated oncogenes or growth factors, as v-ras^Ha^, v-fos or human TGFα in epidermis [34]. In short, there exist some discrepancies about the specific role of p53 in skin cancer, which could be related to inaccurate estimation of p53 inactivation in the experiments involving cre-mediated tissue-specific p53 knock-out, to differences among the experiments regarding the genetic background of the animal models or to differences in the p53 status in dermal cells and the possible existence of cell non-autonomous effects of p53.

Given the generally accepted protective role of p53 in carcinogenesis, our hypothesis is that p53 absence in keratinocytes will cause both spontaneous and chemically induced carcinogenesis. In this work, we have generated mice lacking p53 specifically in keratinocytes (p53^EKO^ mice) and have assessed the efficiency of this deletion. Then, we tried to elucidate the role of epidermal p53 in skin cancer by studying both spontaneous and chemically induced two-stage skin carcinogenesis in mice of homogeneous genetic background. We conclude that p53 in epidermal keratinocytes truly protects against tumor promotion, progression and malignancy in skin, both in chemically-induced and in spontaneous carcinogenesis, thus acting as a *bona fide* tumor suppressor gene, in accordance to the generally admitted role of p53 in other cell types.

## RESULTS AND DISCUSSION

### Efficient p53 inactivation in the skin of p53^fl/fl^; K14-Cre mice

**Figure 1:**
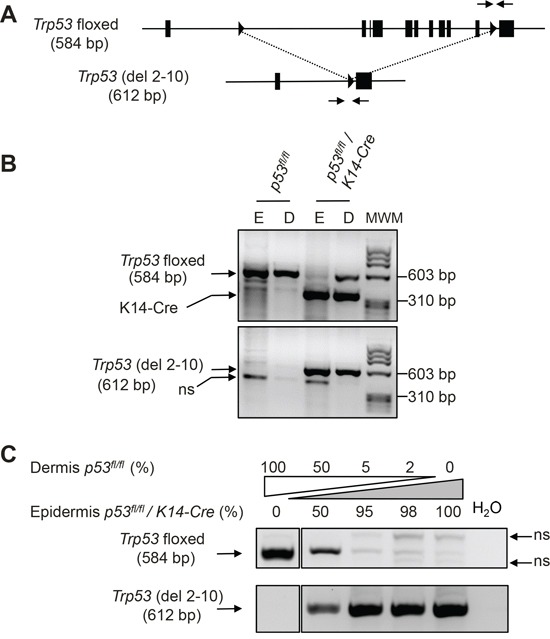
Cre-mediated recombination of floxed *Trp53* alleles affects the majority of epidermal cells **A.** Schematic structure of floxed *Trp53* locus and of the deleted locus lacking exons 2 to 10 (del 2-10) once Cre-mediated recombination has taken place. Triangles represent loxP sites, and the arrows represent the primers used for allele-specific detection. Numbers in parenthesis indicate the length of the amplified fragment in base pairs (bp). **B.** Representative example of allele-specific analysis of p53 in DNA samples obtained from epidermis (E) and dermis (D) of a transgenic mouse with floxed p53 alleles (lanes marked p53^fl/fl^) and from double transgenic mice carrying also the K14-Cre transgene. Primers specific of K14-Cre transgene were also included in the amplification reaction of the upper panel. MWM: DNA molecular weight marker. **C.** Competition PCR of the indicated mixes of DNA form dermis of a non-recombined p53^fl/fl^ mouse and from the epidermis of double p53^fl/fl^/K14-Cre transgenic mice (that presumably have recombined completely the floxed alleles). ns: non-specific bands.

All the cells in the epidermis derive from basal epidermal cells expressing keratin K14. So it is expected that the expression of Cre recombinase from a K14-Cre transgene will lead to the recombination of floxed alleles in the cells of stratified epithelia, including epidermis, although many of these cells actually stop expressing K14 as they proceed through the differentiation process. We generated cohorts of p53^fl/fl^/K14-Cre mice to study the importance of p53 in skin cancer. We did not observe significant deviations between the frequency obtained for each genotype and the expected ratios (not shown). We assessed the efficiency of *Trp53* recombination in epidermis of p53^fl/fl^/K14-Cre mice by PCR analysis using primer combinations able to distinguish between the floxed and the deleted forms of the *Trp53* gene (Figure 1A and 1B). Simultaneous PCR analysis for the Cre transgene and for the floxed form of *Trp53* in DNA from epidermal and dermal tail skin showed the band characteristic of the flallele (584 bp) in both dermis and epidermis in mice lacking the K14-Cre transgene (Figure 1B, upper panel); this band is also clearly detectable in dermal DNA, but not in DNA from epidermis of p53^fl/fl^/K14-Cre mice (Figure 1B, upper panel). Correspondingly, PCR analysis with primers specific for the deleted allele revealed a prevalent 612 bp band in the epidermis of p53^fl/fl^/K14-Cre mice. This band was also detected, although with less intensity, in the dermis of these mice, probably due to the presence of hair follicles expressing the K14-Cre transgene in the dermis (Figure 1B, lower panel). As expected, the 612 bp deletion-specific band was not detected in mice lacking the K4-Cre transgene (Figure 1B, lower panel).

Competition PCRs using mixed DNAs from epidermis of a p53^fl/fl^/K14-Cre mouse (presumably containing mainly recombined *Trp53* alleles) and dermis of a p53^fl/fl^ mouse (containing only floxed *Trp53* alleles) further confirmed that recombination affects to the vast majority of the epidermal cells in mice of the p53^fl/fl^/K14-Cre genotype, as the addition of only 2% or 5% of dermal non-recombined DNA results in a detectable increase in the band indicative of the floxed allele and a reduction in the intensity of non-specific bands (Figure 1C). We reasoned that if the addition of these small amounts of DNA containing floxed p53 alleles is detectable, the initial amount of floxed alleles in the epidermis of p53^fl/fl^/K14-Cre mice must be very low and the recombination efficiency in epidermis must be very high.

From these results, we conclude that p53 deletion is efficient, affecting the majority of epidermal cells of p53^fl/fl^/K14-Cre mice (henceforth, p53^EKO^ mice), and that epidermal p53 deletion does not result in gross deleterious effects over embryonic development or in postnatal life before the age of genotyping and weaning (3 weeks of age).

### p53^EKO^ mice are more susceptible to two-stage skin carcinogenesis than p53^wt^ mice

**Figure 2:**
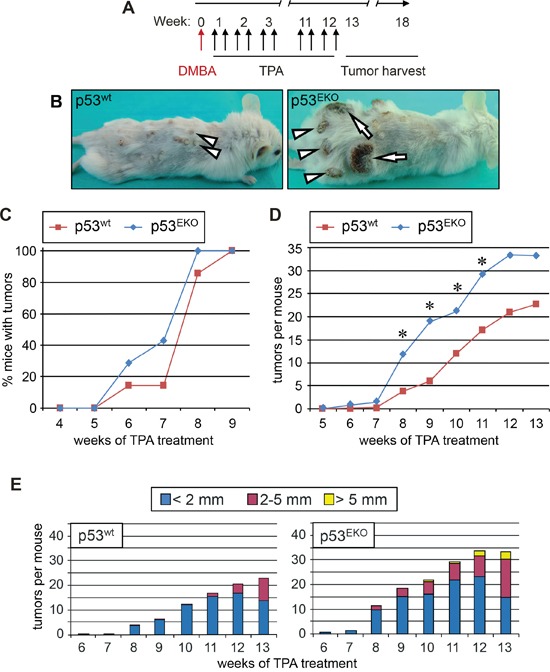
Absence of p53 in epidermis predispose to tumor development in a two-stage chemical carcinogenesis protocol **A.** Timeline of the skin carcinogenesis experiment. 8 to 9 week-old mice were shaved, and three days later treated topically with the carcinogen DMBA. TPA was administered twice a week for 12 weeks. Tumor sampling extended until week 18 in p53^EKO^ mice. **B.** Representative example of a p53^wt^ (left) or p53^EKO^ (right) mouse at week 14. Note the small pedunculated skin tumors with papillomatous aspect in the p53^wt^ mouse and the larger skin tumors in p53^EKO^ mice (arrowheads); in addition, some of the tumors in p53^EKO^ mice are sessile and ulcerated, resembling malignant carcinomas (arrows). **C.** Tumors emerge earlier in p53^EKO^ mice than in p53^wt^ mice when subjected to a DMBA/TPA chemical carcinogenesis protocol. **D.** Time evolution of mean tumor number by phenotypes. p53^EKO^ mice developed more tumors than p53^wt^ mice. Asterisks indicate p value < 0.05 in the t-Student test. **E.** Size distribution of skin tumors in p53^EKO^ and p53^wt^ mice. Note the earlier emergence and the higher number of medium (diameter > 2 mm) and large (diameter > 5 mm) tumors in p53^EKO^ mice in comparison to p53^wt^ mice.

As a first approach for studying the effect of epidermal p53 on cancer, we studied the susceptibility of p53^EKO^ mice to develop skin cancer in a two-stage chemical carcinogenesis protocol; the murine tumors so generated present a mutational landscape highly similar to that found in human SCCs [29]. We performed topical treatment of back skin with the carcinogen DMBA and the hyperplastic agent TPA as indicated in Figure 2A, and registered weekly both the number and size of skin tumors. The experiment finished at week 18 because of the high number and the great size of some of the tumors that arose in p53^EKO^ mice (Figure 2B). Although tumors began to arise on week 6 in both genotypes, and by week 9 all the animals had developed at least one skin tumor, tumors arose faster in p53^EKO^ mice than in p53^wt^ mice, as indicated by the higher percentage of mice bearing tumors in p53^EKO^ compared to p53^wt^ mice in this period of time (Figure 2C). Representative examples of tumor development in p53^wt^ and p53^EKO^ mice at week 14 are shown in Figure 2B. p53^wt^ control mice developed typical exophytic pedunculated cauliflower-like small tumors with gross papillomatous appearance, hard (hyperkeratotic) in consistency, with a dry surface and a poor blood supply (arrowheads in left photograph of Figure 2B). By contrast, tumors developed in p53^EKO^ mice were generally larger, sessile and firmly infiltrated in the dermis (for example, see arrowheads in right photograph of Figure 2B), some of them showing discontinuity of the epidermal barrier, with an ulcerated bleeding surface that did not heal with time; macroscopically, these tumors had a more malignant aspect, resembling skin carcinomas more than papillomas (see arrows in Figure 2B). In addition, although tumors became visible in both genotypes at the same time, p53^EKO^ mice developed more tumors on average than control mice from week 6; these differences were statistically significant between weeks 8 and 11 (Figure 2D). Measurement of tumor size confirmed that lesions from p53^EKO^ mice were larger than those from p53^wt^ mice (Figure 2E). Both the total amount and the percentage of tumors bigger than 2 mm of diameter were greater in p53^EKO^ than in p53^wt^ mice. It is also remarkable the absence of tumors larger than 5 mm of diameter in p53^wt^ mice even at week 14; by contrast, some of the tumors in the population of p53^EKO^ mice reached this size at week 10, and by week 14, 17% of p53^EKO^ tumors (33 out of 196 tumors) had reached this size. In summary, we observed in animals treated with DMBA and TPA that lack of p53 in epidermal cells leads to higher rate of tumor transformation of the skin. These results contrast with the published reduced yield of papillomas in p53 null mice in comparison with p53^wt^ mice [31, 32], and could be explained by differences in the genetic background or perhaps by other differences between the different animal models, such as the different moment of p53 deletion [from the conception in p53 null mice, and around embryonic day 12,5 in our experimental model [35]] or the presence of p53 in dermal cells in p53^EKO^, that do not occur in p53 null mice. In fact, a non-cell-autonomous effect of dermal p53 over tumor growth in xenografts of human tumoral mammary gland or prostate cell lines has been described, although p53 in stromal cells usually hinders tumor cell growth [36, 37]. In sum, our data indicate that, at difference of the described effect of p53 absence in general knock-out mice [31, 32], p53 in epidermal cells actually protects in the first phases of skin tumor development, as p53^EKO^ mice showed increased rates of tumor appearance and tumor growth than mice bearing non-recombined alleles of p53 in their epidermal cells. So we conclude that p53 is relevant in the initiation process of skin carcinogenesis, at least in the context of chemical insults that activates Ras signaling.

### Different skin tumor types emerge in p53^EKO^ mice

**Figure 3:**
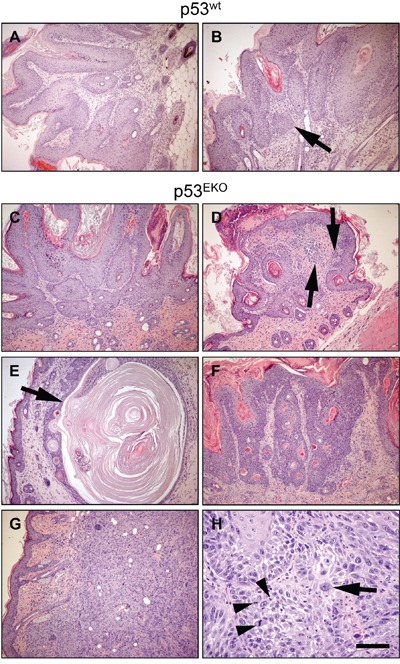
Histological aspect of chemically induced skin tumors Tumors developed in p53^wt^ mice were typical well-differentiated papillomas **A.** that sometimes showed foci of microcarcinoma as groups of epidermal cells deepening in the dermis (**B.**, arrow). **C-H.** Tumors developed in p53^EKO^ mice were histologically more diverse. They ranged from classical papillomas (C) and papillomas with abundant foci of microcarcinoma (D), to tricoepithelioma (E), basosquamous tumors (F) and poorly-differentiated carcinomas (G and H). Scale bars equal 200 microns in A-G and 50 microns in H.

As indicated, p53^EKO^ tumors were frequently larger than those obtained in p53^wt^ mice, indicating a faster growth. Macroscopically, tumors arising in wild type mice were typically exophytic and papillomatous. By contrast, several p53^EKO^ skin tumors had an external appearance suggestive of a more malignant status: they were sessile, with a tendency to infiltrate deeply in the subcutaneous tissue, frequently showing persistent ulceration of the surface. We assessed histologically 18 and 51 tumors from p53^wt^ and p53^EKO^ mice, respectively, collected at weeks 14-18 of the experiment. Microscopically, tumors from p53^wt^ mice were homogeneous, with high level of differentiation and keratinization of the stratified epithelium covering a branched conjunctive tissue stroma, poorly vascularized, featuring the classical histological appearance of squamous papillomas (Figure 3A). Only two of the papillomas showed early signs of malignancy, in the form of small foci of microcarcinoma, composed by nests of epidermal cells with a lack of differentiation of a basal layer invading the stroma (Figure 3B and Table 1). However, p53^EKO^ mice produced more varied tumor histotypes: we found squamous papillomas (Figure 3C) and papillomas that showed higher basal atypia than p53^wt^ papillomas with multiple foci of microcarcinoma (Figure 3D, arrows). Some tumors found in p53^EKO^ mice were tricoepitheliomas, benign lesions from hair follicles composed of cornified cysts, unable to form normal hair (arrow in Figure 3E); others were classified as basosquamous tumors (Figure 3F), lesions derived from hair follicles resembling basosquamous carcinomas, but benign due to the integrity and continuity of the basal membrane. Finally, we also found ulcerative lesions, that correspond to highly malignant skin tumors: infiltrating poorly-differentiated SCCs (18 out of 51 tumors in p53^EKO^ mice, none in p53^wt^ mice), which showed solid areas of increased cellularity infiltrating from the epidermis to the dermis and subcutaneous tissue, composed by keratinocytes with marked cellular pleomorphism, most of them with fibroblastoid (spindle) cell shape, being common the presence of multinucleated cells, cells with aberrant giant nuclei (e.g., arrow in Figure 3H) and mitotic figures (arrowheads in Figure 3H); these features resemble those found in human keratinocytes with p53 loss by means of shRNAs against p53 [38]. These poorly-differentiated SCCs showed a very homogenous solid pattern with little evidence of squamous differentiation and/or keratin deposition (Figure 3G and 3H). Strikingly, all the p53^EKO^ mice included in this study developed at least one poorly-differentiated SCC, precluding the prolongation of the study for the analysis of the evolution of the other tumor types. The incidence of each tumor type is indicated in Table 1. From the data included in Figure 3 and Table 1, we conclude that p53 absence in epidermal cells leads to a marked acceleration of the malignant transformation process of the tumors arisen after treatment with DMBA and TPA. Furthermore, the more varied tumor types observed in p53^EKO^ mice suggests that p53 acts as a tumor suppressor gene in different types of epidermal cells; so lack of p53 would result in different tumor types depending on the degree of commitment or differentiation of the transformed cell type.

**Table 1:** Incidence of tumors and tumor histotype obtained in p53^wt^ and p53^EKO^ mice treated with DMBA and TPA

Type of tumor	p53^wt^ (%)	p53^EKO^ (%)
Squamous papilloma	16 (88,9)	10 (19,6)
Tricoepithelioma	0 (0)	3 (5,9)
Basosquamous tumor	0 (0)	17 (33,3)
Adenosquamous tumor	0 (0)	1 (2,0)
Papillomas with microcarcinomas	2 (11,1)	2 (3,9)
Poorly-differentiated SCC	0 (0)	18 (35,3)
**TOTAL:**	**18**	**51**

### Immunohistochemical analysis indicates increased malignancy of p53^EKO^ tumors

**Figure 4:**
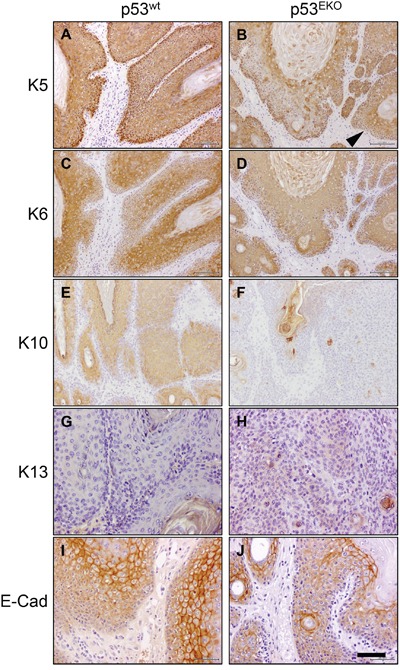
Altered keratin and E-cadherin expression in p53^EKO^ tumors Immunohistochemical analysis of the expression of keratins K5, K6, K10 and K13 and of adhesion molecule E-cadherin in p53^wt^ and p53^EKO^ papillomas. Left row correspond to p53^wt^ tumors, and right row to p53^EKO^ tumors. Scale bars equal 200 microns in **A-F.** and 100 microns in **G-J**.

**Figure 5:**
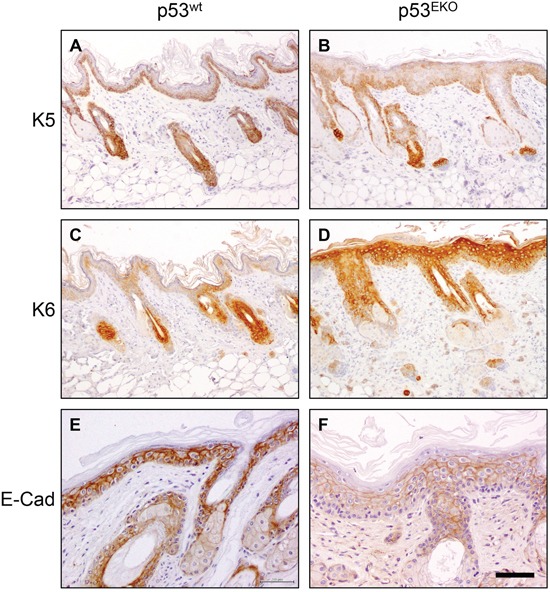
Altered keratin and E-cadherin expression in non-tumoral p53^EKO^ skin Immunochemical analysis of the expression of keratins K5, K6 and E-Cadherin in hyperplastic p53^wt^ and p53^EKO^ back skin. Scale bars equal 200 microns in **A-D.** and 100 microns in **E** and **F**.

In order to define at the molecular level the consequences of p53 absence, we performed an immunohistochemical study of epidermal differentiation markers and of several molecules important in processes related to tumorigenesis, as differentiation, apoptosis or cell cycle regulation (Figures 4 and 5).

p53^wt^ tumors consistently showed stronger staining than p53^EKO^ tumors when assayed with an antibody specific for keratin K5 (Figure 4A and 4B). Moreover, staining indicative of K5 expression was lost in part of the basal epithelium in p53^EKO^ tumors (arrowhead in Figure 4B). By contrast, K5 staining in basal cells of p53^wt^ tumors was stronger and continuous. When using an antibody specific for the epidermal hyperproliferation marker keratin K6, we observed in p53^EKO^ tumors a tendency to express K6 more widely and in more layers than p53^wt^ tumors (Figure 4C and 4D). The expression of the normal epidermal differentiation marker K10 was higher in p53^wt^ than in p53^EKO^ tumors, indicating a lower differentiation degree in p53^EKO^ tumors (Figure 4E and 4F). Conversely, p53^wt^ tumors did not express keratin K13, a protein associated to epidermal carcinoma progression, but this protein was actually detected, although faintly, in some p53^EKO^ tumors (Figure 4G and 4H). Interestingly, E-cadherin, an adhesion protein downregulated in cancer progression and metastasis, is expressed at higher level in p53^wt^ papillomas than in p53^EKO^ papillomas (Figure 4I and 4J), indicating a stronger tendency to malignancy in papillomas lacking p53 than in papillomas grown in p53^wt^ mice. Taken together, the changes in expression of keratins and E-cadherin in skin tumors indicate molecularly that p53 absence leads to the development of papillomas prone to malignancy, as they express higher amounts of keratins associated to malignant transformation, and less amounts of E-cadherin. We next studied if these alterations in differentiation and adhesion markers were already present in non-tumoral skin. With this aim, we treated p53^EKO^ and p53^wt^ mice twice every other day with TPA, in order to induce a hyperplastic state in the skin that facilitates the detection of differences in these proteins. We could distinguish differences in the expression of K5, K6, and E-cadherin in both genotypes, independent of tumoral transformation: so hyperplastic p53^EKO^ skin showed fainter and irregular K5 staining in the basal layer, more intense and wider staining for K6 and milder E-cadherin staining than hyperplastic p53^wt^ skin (Figures 5A–5F). All these molecular changes found in tumors and in non-tumoral skin are known to be important in the process of malignant transformation and in the development of invasion and metastasis capabilities by epithelial tumor cells [39, 40].

**Figure 6:**
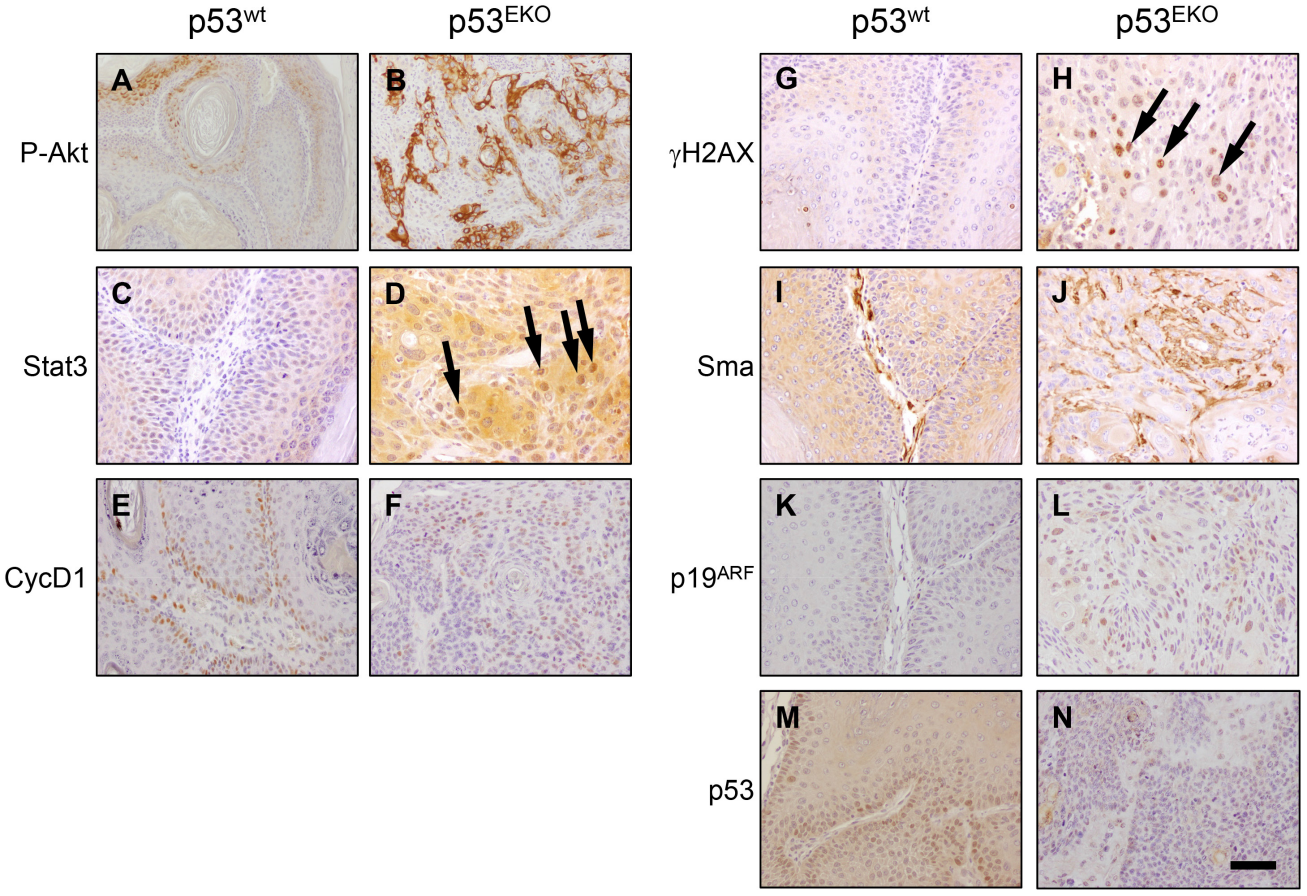
Immunohistochemical analysis of signaling proteins in p53^wt^ and p53^EKO^ tumors Photographs are representative fields showing the expression of proteins relevant in carcinogenesis: P-Akt, Stat3, CycD1, γH2AX, Sma, p19^ARF^ and p53. Scale bars equal 200 microns in **A** and **B.**, and 100 microns in **C-N**.

We also analysed the expression of proteins implicated in cell proliferation, DNA damage and angiogenesis in p53^wt^ papillomas and p53^EKO^ SCCs (Figure 6). Akt signaling pathway is important in the regulation of cell survival, cell cycle progression, metabolism, angiogenesis and in epithelial carcinogenesis [41]. Interestingly, Akt seems to be activated in p53^EKO^ tumors, as indicated by the widespread staining observed for activated P-Akt in these tumors (compare Figure 6A and 6B). We also found more intense staining for Stat3, a mediator of proliferation and antiapoptotic signals, in p53^EKO^ tumors than in p53^wt^ tumors (compare Figure 6C and 6D); interestingly, Stat3 is detected in nuclei in p53^EKO^ samples (arrows in Figure 6D), indicating activation of this transcription factor, a feature widely found in numerous malignancies [42, 43]. It is interesting to note that a regulation of Stat3 by p53 has been described, so that wt p53 (but not mutant forms of p53) reduces Stat3 phosphorylation at least in breast, ovarian and prostate cells [44]. Cyclin D1, a regulator of cell cycle entry, is found in most of the nuclei of the basal layer in p53^wt^ tumors (Figure 6E); interestingly, p53^EKO^ tumors showed Cyclin D1 staining also in other cell layers (Figure 6F), indicating higher proliferative potential. Consistent with the role of p53 as a pivotal molecule in the maintaining of genetic stability, p53^EKO^ tumors showed increased staining for γH2AX, a marker of DNA double stranded breaks [45] (Figure 6G and 6H); this result indicate that p53^EKO^ tumors accumulate more DNA damage, a feature associated to malignant transformation. We also analyzed the expression of α-smooth-muscle-actin (Sma), a marker of perivascular cells of the blood vessels and of tumoral angiogenesis; its expression is increased in p53^EKO^ tumors (Figure 6I and 6J), indicating an increased blood supply and consequently growth ability in these tumors. p19^ARF^ is a protein that mediates the accumulation of p53 in cells subjected to oncogenic signals, and its level is increased in cells lacking p53 [46]. When the expression of p19^ARF^ was analyzed, we found consistently an increase in the p19 nuclear staining in p53^EKO^ tumors (Figure 6K and 6L). Finally, tumor cells in p53^EKO^ mice did not show immunoreactivity against an antibody specific for p53 protein (Figure 6M and 6N), indicating that these lesions are actually formed from epidermal cells lacking p53.

**Figure 7:**
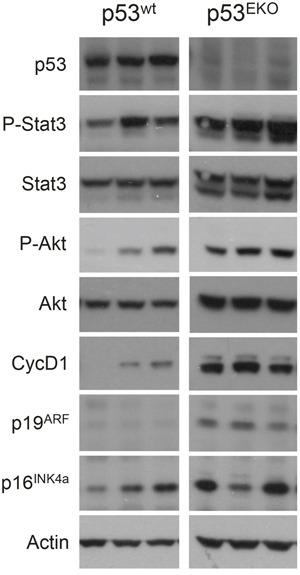
Expression of signaling proteins and cell cycle regulators in p53^wt^ and p53^EKO^ tumors Western blot analyses of the indicated proteins in three different tumors from p53^wt^ and p53^EKO^ mice are shown.

Western blot analysis of p53^wt^ and p53^EKO^ tumors further confirmed the results obtained in the immunohistochemical analysis (Figure 7). Tumors originated in p53^EKO^ mice showed a marked decrease in the band specific for p53, as expected. Our western blot analysis also showed over activation of Stat3 and Akt pathways and higher levels of CyclinD1 in p53^EKO^ tumors, in concordance with the results shown in Figure 6. The tumor suppressor gene p19^ARF^ is also consistently overexpressed in p53^EKO^ tumors, probably as an attempt of inhibiting tumor growth in epidermal cells lacking p53 by p53-independent mechanisms [32]. Interestingly, these changes in expression are not seen for p16^INK4a^, indicating the differential roles of these two tumor suppressor proteins in skin cancer. We have not detected differences in mTOR signaling (measured as phosphorylation of the ribosomal protein S6) in tumors or in non-tumoral skin of p53^wt^ and p53^EKO^ genotypes (not shown).

In summary, the expression analysis performed in tumors from p53^wt^ and p53^EKO^ mice highlight the protective role of p53 in skin cancer, as lack of p53 in epidermal cells results in tumors with activated pro-tumoral signaling pathways and with changes in other molecules that result in more malignant tumors.

### Lack of p53 in epidermal cells leads to spontaneous tumor formation

In an attempt to determine the importance of p53 in spontaneous carcinogenesis, independent of the Ras mutations induced by DMBA/TPA treatments, we studied tumor formation in aging mice.

**Figure 8:**
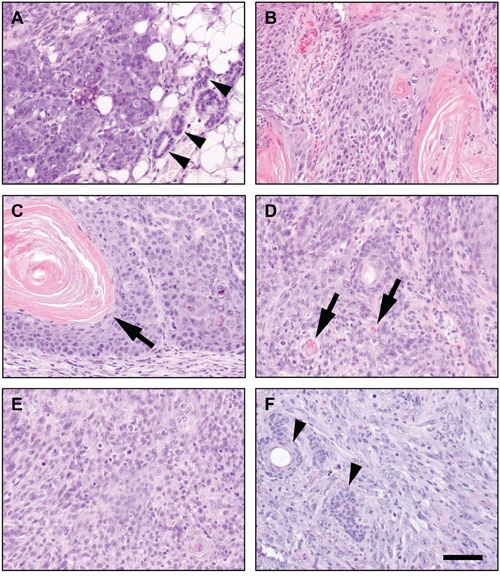
Spontaneous epithelial tumors in mice lacking p53 in stratified epithelial cells Representative examples of spontaneous tumoral lesions arising in aging p53^EKO^ mice. **A.** Mammary gland carcinoma. Arrowheads indicate non-tumoral mammary ducts. **B.** SCC of oral epithelia. **C.** Benign papilloma in skin. Arrow marks a keratin pearl. Note the well distributed basal cell layer, characteristic of benign skin tumors. **D.** Well-differentiated SCC of skin. Small squamous differentiation foci around keratin deposits (pearls) are formed in several parts of the tumor (arrows). **E.** Poorly-differentiated skin SCC, formed by solid masses of keratinocytes showing scattered foci of squamous differentiation. **F.** Spindle undifferentiated SCC, containing abundant fibroblastoid spindle-shaped cells that have lost the epithelial morphology. Remnants of hair follicles are still visible (arrowheads). Scale bars equal 100 microns.

Absence of p53 is able to cause spontaneous tumor formation in different cell types [15], but not in all of them, as enterocytes lacking p53 do not give rise to spontaneous intestinal tumors [33]. We generated a cohort of 21 male- and 16 female-p53^EKO^ mice, and analyzed both tumor emergence and the histological type of the tumors arisen. Mice were maintained in the study until they were 14 months old or had visible tumors larger than 5 mm in diameter. A significant number of the p53^EKO^ mice (11 out of 37) succumbed by unknown pathologies before the end of the experiment; from the rest of the animals, the majority of them (20 out of 26) developed epithelial tumors before the age of 14 months. The histological classification of these tumors is indicated in Table 2, and representative examples are shown in Figure 8. Spontaneous carcinogenesis in wild-type mice is a very rare event in the time period analyzed. We observed 38 different tumors in these 20 p53^EKO^ animals, that arose mainly in skin (32 tumors), but also in other epithelial tissues expressing K14 that presumably suffered also Cre-mediated p53 inactivation, as mammary gland and oral epithelia (3 tumors in each one; Figure 8A and 8B). It is remarkable that, besides some benign tumors (papillomas, keratoacanthomas and basosquamous tumors; e.g., Figure 8C) the majority of the tumors analyzed were malignant, ranging from relatively well differentiated SCCs (Figure 8D) to poorly-differentiated SCCs (Figure 8E) and highly aggressive carcinomas formed by undifferentiated spindle cells (Figure 8F). In summary, mice lacking p53 in stratified epithelia have a shortened lifespan, with a tendency to develop tumoral lesions in epidermis but also in other epithelial tissues, even without the existence of chemically induced Ras mutations; furthermore, these tumors easily become malignant, being relatively frequent the emergence of poorly-differentiated SCCs and spindle SCCs.

**Table 2:** Classification of spontaneous epithelial tumors in p53^EKO^ mice

Tissue	Type of tumor	Number of tumors
Oral epithelia	Carcinoma	3
Mammary gland	Carcinoma	3
	Benign tumor	9
	Differentiated SCC	12
Skin	Poorly-differentiated SCC	5
	Spindle undifferentiated SCC	6

The skin tumors were classified histologically as benign (squamous papillomas, keratoacanthomas and basosquamous tumors), differentiated and poorly-differentiated SCCs, and spindle cell carcinomas, containing highly transformed fusiform cells.

Taken together, the results presented in this report indicates that epidermal loss of p53 function facilitates both papilloma emergence and their growth in two-stage skin chemical carcinogenesis, and also increases the rate of malignant transformation of these tumoral lesions. The increased diversity of epidermal tumor types obtained in p53^EKO^ mice indicates a tumor suppressive role of p53 in a variety of epidermal cell types. p53 absence in keratinocytes also leads to the spontaneous development of highly malignant poorly-differentiated SCCs in skin and other stratified epithelia, without the need of induced mutations in Ras genes that occurs after DMBA treatment. In summary, our results show that p53 absence increases initiation, growth and malignancy of skin tumors, and that its function is truly and totally protective in skin carcinogenesis, confirming a role for p53 as a genuine tumor suppressor gene in skin carcinogenesis.

## MATERIALS AND METHODS

### Mice and treatments

Animal work was conducted following protocols approved by our institutional Ethical Committee for Animal Experiments and according European, Spanish and local regulations; the experiments included in this publication are under the permit number BME 02/10 of the Ethical Committee for Animal Experiments at CIEMAT. All efforts were made to minimize suffering of the animals employed. Mice were maintained on autoclaved standard rodent chow and water *ad libitum* and kept under a 12 h light −12 h dark cycle. Floxed *Trp53* mice and K14-Cre mice have been described previously [17, 47]. For obtaining mice lacking p53 in epidermis, we crossed p53^wt/fl^; K14-Cre female mice with p53^wt/fl^ males. In mice inheriting both the K14-Cre transgene and floxed alleles of *Trp53*, Cre activity would delete exons 2 to 10 of *Trp53* in epidermis and other cell types expressing the K14-Cre transgene [47] (Figure 1A). This recombined allele is unable to produce a functional protein, as it lacks part of the transcriptional activation domain, the DNA-binding domain and the tetramerization domain of p53. We selected for the carcinogenesis studies p53^fl/fl^; K14-Cre mice (referred in this work as p53^EKO^) and p53^wt/wt^ littermates as control mice (referred in this work as p53^wt^). All the mice used in the carcinogenesis studies were of the same mixed genetic background (50% hybrid C57BL6JxDBA2J and 50% FVB). Cohorts of seven mice of each genotype were subjected to a cutaneous two-stage chemical carcinogenesis protocol. In this protocol, mice were shaved using a hair clipper and treated three days later with a single topical application of 100 μg of 7,12-dimethylbenz[a]anthracene (DMBA, Sigma-Aldrich, reference D3254) dissolved in 200 μl acetone (week 0). Seven days after DMBA application, 5 μg of 12-O-tetradecanoylphorbol 13-acetate (TPA, Sigma-Aldrich, reference P1585) in 200 μl acetone was applied topically twice a week for 12 weeks. The number and size of tumors per mouse was recorded weekly. p53^EKO^ mice were euthanized when the tumors had a diameter greater than 1 cm or at week 18, due to humanitarian reasons. Four p53^wt^ mice were time-paired sacrificed between weeks 13 and 18, and the rest were sacrificed between weeks 35 and 40, in order to get lesions of enough size to permit the analysis by western blot. Samples were processed for histological and western blot analyses, and the mice genotypes were confirmed by reanalyzing new DNA samples taken postmortem.

For spontaneous carcinogenesis, mice were monitored for tumor development for 14 months. Mice showing tumors or with obvious signs of disease were euthanized for necropsy and histological analysis.

For the study of non-tumoral hyperplastic skin, three p53^EKO^ mice and the same number of p53^wt^ mice were shaved and topically treated twice with 5 μg of TPA or vehicle at days 3 and 5 after shaving.

### Genotyping and PCR analysis

DNA was obtained from tail biopsies of 2 week old-mice or from samples taken postmortem. Mice were genotyped by PCR. Primers used for determination of the *Trp53* status (i.e.: floxed, deleted or wild type) and for detecting the presence of the K14-Cre transgene are indicated in [47].

### Histology and immunohistochemistry

Mouse tumors were dissected and immediately fixed in 10% buffered formalin or 70% ethanol and embedded in paraffin. 5 μm-thick sections were used for H&E staining or immunohistochemical preparations. Most of the tumors were fixed and classified by morphology after sectioning and staining with H&E. The antibodies used in the immunohistochemical analysis were against p53 (NCL-p53-CM5p, Novocastra, Leica Biosystems, NewCastle, UK); keratin K5 (PRB-160P), K10 (PRB-159P) (Covance, San Diego, CA); keratin K13 (ab6112), p19^ARF^ (ab80) (Abcam, Cambridge, UK); smooth muscle actin (C6198, Sigma-Aldrich, St Louis, MO); Cyclin D1(RM-9104-RQ, Thermo Fisher Scientific, MA, USA); Phospho-Akt1(ser 473) (9277), Stat3 (4904) (Cell Signaling Technology, Danvers, MA, USA) and BrdU (11170376001, Roche, Mannheim, Germany). Immunoreactivity was revealed using an ABC avidin-biotin-peroxidase system and ABC substrate (Vector Laboratories), and the sections counterstained slightly with haematoxylin. Control experiments omitting the primary antibody gave no signals.

### Western blot

In those tumors that were large enough, we froze part of the tumor in liquid nitrogen at the moment of the sacrifice for western blot analysis. Whole-cell protein extracts were subjected to SDS/PAGE using standard techniques. Protein content was determined by the Bradford colorimetric protein assay (BioRad Laboratories; Hercules, CA; USA). The antibodies used in western blots were against p53 (NCL-p53-CM5p, Novocastra, Leica Biosystems, NewCastle, UK); Stat3 (phospho tyr705; 9131), Stat3 (4904); Akt (phospho ser473; 4058) (Cell Signaling Technology, Danvers, MA, USA); p19^ARF^ (ab80) (Abcam, Cambridge, UK); Akt1/2 (sc-1619), p16^ink4a^ (sc-1207), Cyclin D1(sc-753), and actin (sc-1616) as a loading control (Santa Cruz Biotechnology, Santa Cruz, CA, USA).

### Statistical analysis

Data are expressed as mean ± SEM. P values were determined by using the unpaired, two-tailed Student t test. P values < 0.05 were considered significant.

## Abbreviations

bp: base pairs
H&E: haematoxylin and eosin
Tg: transgenic
fl: floxed and flanked by loxP sites
SCC: Squamous cell carcinoma
SpCC: Spindle cell carcinoma
wt: wild type.
