# Correction: Protective role of p53 in skin cancer: Carcinogenesis studies in mice lacking epidermal p53

**DOI:** 10.18632/oncotarget.16592

**Published:** 2017-03-27

**Authors:** Angustias Page, Manuel Navarro, Cristian Suarez-Cabrera, Josefa P. Alameda, M. Llanos Casanova, Jesús M. Paramio, Ana Bravo, Angel Ramirez

Present: Due to an error in grant support.

Correct: The updated grant support are given below. The author sincerely apologizes for this error.

Original article: Oncotarget. 2016; 7(15):20902-18. doi: 10.18632/oncotarget.7897.

